# Effect of Inoculation Volume on a Mouse Model of Influenza Virus Infected with the Same Viral Load

**DOI:** 10.3390/vaccines13020173

**Published:** 2025-02-12

**Authors:** Yali Sun, Yuwei Wei, Xuelian Han, Yuan Wang, Qi Yin, Yuhang Zhang, Tiantian Yang, Jiejie Zhang, Keyu Sun, Feimin Fang, Shuai Zhang, Kai Yuan, Min Li, Guangyu Zhao

**Affiliations:** 1 Public Health School, Mudanjiang Medical University, Mudanjiang 157011, China; 18852551076@163.com (Y.S.); ytt_tjutcm@163.com (T.Y.); s1010457183@163.com (K.S.); ykmarceau@163.com (K.Y.); 2 State Key Laboratory of Pathogen and Biosecurity, Academy of Military Medical Sciences, Beijing 100071, China; weiyuweii9872@163.com (Y.W.); hanxuelian@bmi.ac.cn (X.H.); wangyuan1@bmi.ac.cn (Y.W.); yinqi@bmi.ac.cn (Q.Y.); yhang7710@163.com (Y.Z.); zhangjiejie202202@163.com (J.Z.); fgh66666@163.com (F.F.); shuaiz2024@stu.ahau.edu.cn (S.Z.); 3 School of Basic Medical Sciences, Anhui Medical University, Hefei 230032, China; 4 School of Life Sciences, Anhui Agricultural University, Hefei 230036, China; 5 Laboratory of Advanced Biotechnology, Academy of Military Medical Sciences, Beijing 100071, China

**Keywords:** influenza virus, mouse model, inoculation volume, viral load, infection, influenza vaccine evaluation

## Abstract

Background: Influenza is a highly contagious respiratory disease that poses significant health and economic burdens. Mice are commonly used as animal models for studying influenza virus pathogenesis and the development of vaccines and drugs. However, the viral volume used for nasal inoculation varies substantially in reported mouse influenza infection models, and the appropriate viral dose is crucial for reproducing experimental results. Methods: Mice were inoculated with mouse lung-adapted strains of influenza virus A/Puerto Rico/8/34 (H1N1) via intranasal administration of 10 μL, 20 μL, and 40 μL at doses of 200 plaque-forming units (PFU) and 2000 PFU. This study investigated the impact of varying viral inoculum volumes on murine outcomes at identical doses and assessed the disparities across diverse dosage levels. Results: Regarding weight change trajectories, mortalities, lung tissue viral titers, and pathological manifestations, the group that received the 40 μL inoculation volume within the low-dose infection mice (200 PFU) manifested a statistically significant divergence from those inoculated with both the 10 μL and 20 μL volumes. Within the context of high-dose infections (2000 PFU), groups that received inoculation volumes of 20 μL and 40 μL exhibited marked disparities when compared to those receiving the 10 μL volume. Conclusions: Disparities in inoculation volume, even under uniform infection dosages, engender differential outcomes in pathogenicity. Of particular note, the viral replication efficacy at a 20 μL inoculation volume demonstrates conspicuous fluctuations across diverse infection dose regimens.

## 1. Introduction

Influenza, a highly infectious respiratory disease caused by the influenza virus, poses significant health and economic burdens globally. According to the World Health Organization, millions of people are infected with influenza annually, and hundreds of thousands die of the disease [1,2]. Therefore, studying influenza pathogenesis and developing vaccines and drugs for its prevention and treatment are crucial, necessitating suitable animal models. Common laboratory animals used in influenza studies include mice, ferrets, and Guinea pigs [3,4,5]. Ferrets, one of the earliest models for studying influenza virus pathogenesis, have limitations such as a lack of inbred lines, large size, high cost, and difficulty in procurement [5,6]. Guinea pigs are commonly used to study the respiratory transmission mechanism of the influenza virus, but because of the lack of clinical manifestations of influenza virus infection, they are not suitable for studying influenza virus pathogenicity [7]. Therefore, mice remain the most commonly used animal models for studying influenza virus pathogenesis and developing vaccines and drugs, with C57BL/6 and BALB/c inbred strains being the most commonly used [3]. However, there are significant differences in the distribution of sialic acid receptors in the respiratory tracts of mice and humans [8]. In order to obtain influenza virus strains with high pathogenicity in mice, it is necessary to conduct adaptive passages of influenza viruses in mice. The resulting adapted strains have an infectious capability in mice that is close to that of human influenza viruses in humans [9]. Therefore, in this study, BALB/c strain mice and A/Puerto Rico/8/34(H1N1) mouse lung-adapted strains of influenza virus were selected to construct a mouse model of influenza virus infection.

The primary transmission modes of the influenza virus among humans are aerosol, droplet, and contact [10]. To construct an animal model of influenza viral infection, a mode similar to that of human transmission should be selected. Currently, the commonly used viral infection methods are nasal drip and aerosol infection [11,12]. Aerosol infections require a supporting device, and the resulting aerosol dose cannot ensure uniform infection in all animals. Therefore, nasal drops remain the preferred viral infection method because of their simplicity, accuracy, and controllability [13]. However, there is variation in the documented volumes of viruses used for nasal drops. The reported volumes for nasal drop infections in mice include 15 μL [14], 20 μL [15,16,17,18], 25 μL [19,20], 30 μL [21,22], 40 μL [23,24], 50 μL [13,25,26,27,28], and 100 μL [29]. Among these, 50 μL is the most common volume. And studies have shown that, compared with 50 μL, there is no further increase in the relative lung distribution of the inoculum when 75 μL is instilled [30]. However, another study suggests that intranasal infection with 50 μL of nasal drops in mice during the experiment can cause respiratory difficulties or even death [31]. Therefore, in order to reduce adverse events, and considering ethical and scientific factors, the volume of virus for intranasal infection should be as low as possible below 50 μL. Previous studies have shown that administering 25 μL of viral volume at the same infective dose to mice can significantly reduce the incidence in mice [31], but the variation in this effect at different doses remains unknown. Therefore, to evaluate the effect of different influenza viral infection volumes administered via nasal drops on the effectiveness of viral infection in mice, this study selected a mouse lung-adapted strain of influenza virus A/Puerto Rico/8/34(H1N1). The virus was administered at two doses, 200 PFU and 2000 PFU. The study analyzed the effects of different viral infection volumes on the effectiveness of virus infection in mice at the same dose and the variations of these effects across different doses, providing a reference for selecting appropriate viral infection volumes to establish mouse models for influenza virus studies.

## 2. Materials and Methods

### 2.1. Ethical Statement

All animal experimental protocols were reviewed and approved by the Institutional Animal Care and Use Committee of Academy of Military Medical Sciences (Permit Number: IACUC-IME-2022-045).

### 2.2. Viruses and Cells

Madin–Darby canine kidney (MDCK; ATCC number:CCL-34) cells were cultured in Dulbecco’s modified Eagle’s medium (DMEM) supplemented with penicillin–streptomycin, 4-(2-hydroxyethyl)-1-piperazine vinyl sulfonic acid (HEPES) buffer, and 10% fetal bovine serum. The influenza strain A/Puerto Rico/8/34(H1N1) (preserved in our laboratory) mouse lung-adapted strain was amplified in specific pathogen-free (SPF) chick embryos at 35 °C for 48 h, and the chick embryo allantoic fluid containing the virus was collected. The virus titer was determined to be 3 × 10^8^ PFU/mL using a plaque assay for MDCK cells, and the virus stock was stored at −80 °C prior to use.

### 2.3. Animals

In total, 165 seven-week-old SPF-grade female BALB/c mice weighing 18–20 g were purchased from SPF (Beijing, China) Biotechnology Co., LTD. (SYXK [Beijing] 2020-0051). Before infection, mice were anesthetized via intraperitoneal injection of pentobarbital sodium (30 mg/kg body weight) [32,33]. In animal experiments, measures to prevent cross-contamination between groups included treating the low-dose infection group before the high-dose infection group, treating the same dose infection group from low-volume to high-volume infection group, and disinfecting gloves and replacing scissors and tablecloth after treating each group.

### 2.4. Mouse Influenza Virus Infection Experiment

A total of 150 seven-week-old, specific pathogen-free (SPF) female BALB/c mice were randomly distributed into six groups, each comprising 25 individuals. Within this cohort, half of the mice underwent intranasal administration of 200 plaque-forming units (PFU) of the mouse lung-adapted influenza A/Puerto Rico/8/34 (H1N1) strain, while the remaining mice were inoculated with a higher dose of 2000 PFU. Mice in each group were intranasally inoculated in a supine position, with a pipette of appropriate volume slowly and evenly dripping the virus into the left naris. The experiment was structured such that for every set of three groups infected with either 200 or 2000 PFU, distinct inoculum volumes of 10 μL, 20 μL, and 40 μL were employed, respectively, to assess dose–response relationships.

In each experimental group, ten out of the twenty-five mice were subjected to continuous observation for a period of 14 days after viral inoculation. During this period, the body weight changes of mice in each group were recorded daily, and mice that lost more than 20% of their original body weight were regarded as dead, and survival curves were plotted. Concurrently, the remaining mice within each group were humanely euthanized at designated intervals of 1, 3, and 5 days post-infection (Table 1). Upon necropsy, lung tissues were harvested, with the left lung lobe being reserved for subsequent histopathological assessments. The residual lung tissue was processed into a 10% (*w*/*v*) homogenate employing Dulbecco’s modified Eagle’s medium (DMEM) as the diluent, facilitating the quantification of viral titers present within the lung parenchyma.

**Table 1:** Experiment setup with group divisions for mouse influenza virus infection.

Infectious Dose (PFU)	Infectious Volume (μL)	Sampling Group (*n* = 90)			Observation Group (*n* = 60)
		Dpi1	Dpi3	Dpi5	
200	10	5	5	5	10
	20	5	5	5	10
	40	5	5	5	10
2000	10	5	5	5	10
	20	5	5	5	10
	40	5	5	5	10

Abbreviations: Dpi, days post-infection.

### 2.5. Detection of Virus Titers in Lung Tissue

Virus titers in lung tissue were determined using tissue culture infective dosimetry (TCID_50_). The filtered lung homogenate was diluted 10-fold with viral maintenance solution. The 96-well culture plates containing single-layer MDCK cells were removed from the incubator, and the cell culture medium was aspirated from each well. The plates were washed twice with sterile PBS. Diluted lung homogenate was added to MDCK cells and incubated for 2 h. The virus solution was discarded, and 200 μL of viral maintenance solution was added to the incubator for 72 h. Cytological changes were observed under a microscope. The virus titer was calculated using the Reed–Muench method [34] and expressed as log_10_50% tissue culture infective dose (TCID_50_) g of lung tissue.

### 2.6. Lung Histopathology

The left lung lobe was fixed with 4% paraformaldehyde, paraffin-embedded, sliced, and stained with hematoxylin and eosin, and the images were scored by double-blind microscopy.

### 2.7. Fluorescence Experiment of Cy7.5 Dye in Mice

Fifteen 7-week-old SPF female BALB/c mice were randomly divided into three groups of five mice per group. Mice were anesthetized with isoflurane inhalation and inoculated with Cy7.5 dye via nasal drops at doses of 10 μL, 20 μL, and 40 μL. Ten minutes after inoculation, the mice were anesthetized via intraperitoneal injection of pentobarbital sodium (30 mg/kg body weight) [32,33], and hair was removed from the neck and chest. Mice were imaged in vivo 20 min after inoculation, and after imaging, airway tissues such as the trachea and lungs were dissected for tissue-level imaging.

### 2.8. Statistical Analysis

All statistical analyses were performed using GraphPad Prism (version 7.00; GraphPad Software, Inc., La Jolla, CA, USA). Given that the data are normally distributed, Tukey’s post hoc two-way ANOVA with the default Geisser–Greenhouse correction for homogeneity of variance. This analysis aimed to compare the changes in body weight among groups with different infection volumes over increasing days of infection, all at the same infection dose. Additionally, Tukey’s post hoc one-way ANOVA was used to compare the viral titer results among groups with different infection volumes at the same infection dose at specific time points. Meanwhile, under the same inoculation dose, Tukey’s post hoc one-way ANOVA was also performed on the fluorescence intensity results of each group with varying inoculation volumes of Cy7.5 dye.

## 3. Results

### 3.1. Impact of Inoculation Volume on Pathogenicity in Mice with Consistent Influenza Virus Dosage

**Figure 1:**
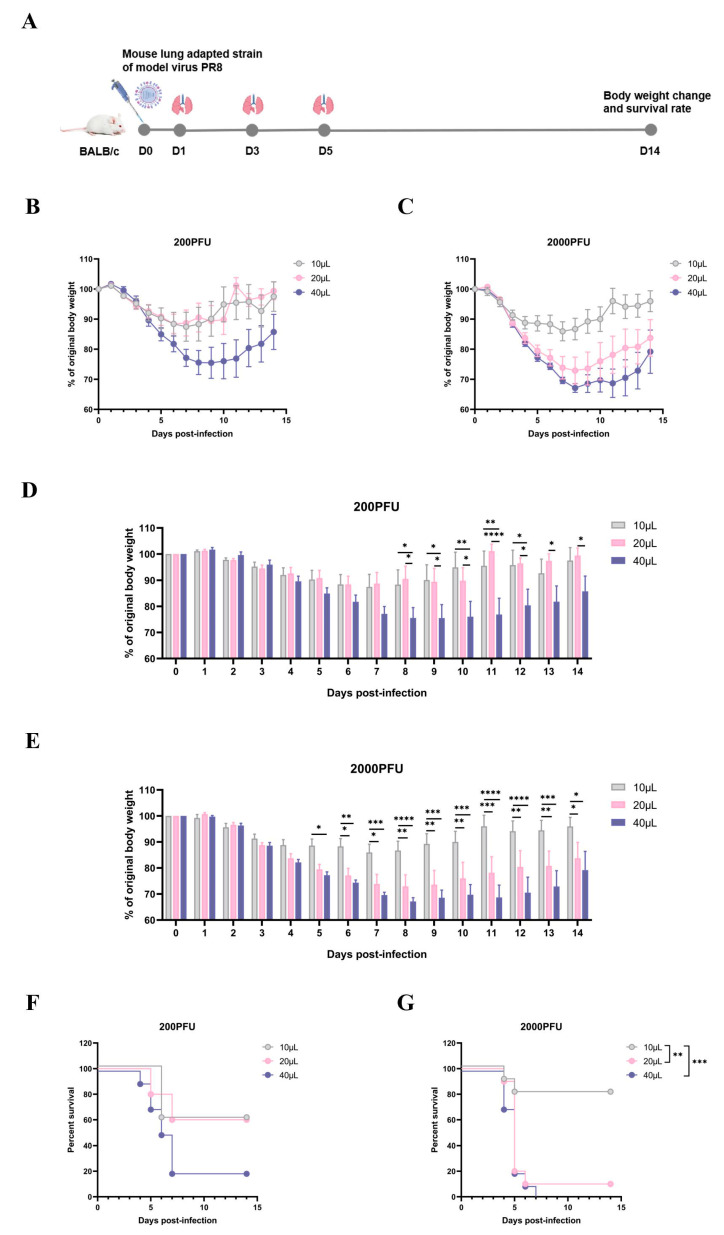
Effect of influenza virus with different infection volumes on pathogenicity in mice at the same infection dose. Mice were infected nasally with 200 and 2000 PFU lung-adapted strains of model virus A/Puerto Rico/8/34(H1N1), with infection volumes of 10 μL, 20 μL, and 40 μL. Ten mice were infected with each volume. During the 14-day observation period, the weight of mice in each group was recorded daily, and those with a body weight reduction exceeding 20% of their original weight were considered dead and documented. Weight reduction is expressed as a percentage of the original weight. (**A**) Mouse challenge diagram. (**B**–**E**) Changes in body weight of mice in low-dose and high-dose infection groups within 14 days. (**F**,**G**) Survival curves of mice in low-dose and high-dose infection groups. * *p* < 0.05, ** *p* < 0.01, *** *p* < 0.005, **** *p* < 0.001.

Following the experimental design (Figure 1A), sixty 7-week-old specific pathogen-free (SPF) female BALB/c mice were randomly segregated into six distinct groups, each comprising ten individuals. Viral infection was administered intranasally, with two dosage regimens established: a low-dose group exposed to 200 plaque-forming units (PFU) and a high-dose group subjected to 2000 PFU. Within these categories, three incremental inoculum volumes—10 μL, 20 μL, and 40 μL—were tested to explore dose-dependent effects. During the stringent 14-day observation period post-infection, the body weight of each mouse was measured daily, and those experiencing a weight loss exceeding 20% were considered dead and recorded accordingly.

The data revealed nuanced patterns in weight dynamics across both dosage groups. In the lower-dose stratum, mice receiving 10 μL and 20 μL viral doses exhibited analogous weight fluctuation trajectories, diverging significantly from those challenged with a 40 μL inoculum (Figure 1B,D). Conversely, within the higher-dose framework, a clear escalation in weight loss was evident in tandem with the increment of infection volumes. Notably, the weight alteration trends for mice inoculated with 20 μL and 40 μL of virus starkly contrasted with those administered the minimal 10 μL dose (Figure 1C,E).

Regarding mortality outcomes, both experimental cohorts witnessed fatalities commencing on the fourth day post-inoculation. Nonetheless, the onset of deaths in the low-dose group manifested a delayed pattern correlated with reduced viral loads when juxtaposed against the accelerated mortality observed in the high-dose counterparts. Furthermore, within the latter group, a direct correlation was observed between increased viral infection volumes and elevated mortality rates. Specifically, the mortality rate for mice infected with 20 μL and 40 μL viruses was notably higher than that for those infected with 10 μL (Figure 1F,G), underscoring the dose-dependent nature of these outcomes.

### 3.2. Replication Dynamics of Influenza Virus in Mouse Lungs Following Inoculation with Identical Viral Dosage at Varied Volumes

**Figure 2:**
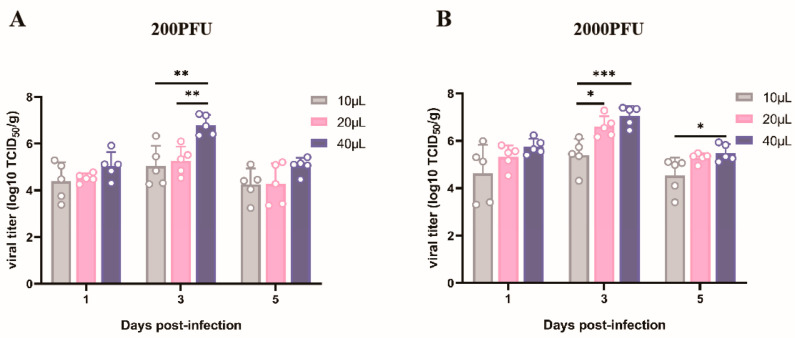
Viral replication of influenza virus strain A/Puerto Rico/8/34(H1N1) mouse lung-adapted strain in mouse lung tissue. At 1 day, 3 days, and 5 days post-infection, five mice per group were euthanized in batches. The left lung lobe tissues were collected and fixed with 4% paraformaldehyde, and the remaining lung tissue samples were weighed and homogenized. Viral titers in lung homogenates were determined by tissue culture infective dosimetry (TCID_50_). (**A**) Viral titers in mouse lung tissue of 200 PFU in the low-dose infection group. (**B**) Viral titers in mouse lung tissue of 2000 PFU in the high-dose infection group. Virus titers in lung tissue were expressed as TCID_50_/g * *p* < 0.05, ** *p* < 0.01, *** *p* < 0.005.

To compare the dynamics of viral replication in mouse lung tissue following inoculation with an equivalent dose of virus administered in varying inoculation volumes, lung tissues harvested from sacrificed mice at 1, 3, and 5 days post-inoculation were processed into homogenates for the determination of lung viral titers. The findings indicated that irrespective of the inoculation dose, whether it was the lower 200 PFU or the elevated 2000 PFU, viral replication reached its zenith at day 3 post-infection. Within the context of the low-dose group receiving 200 PFU of virus, comparable peak viral titers were observed between the 10 µL and 20 µL inoculation volumes. Conversely, a marked disparity emerged when comparing these to the peak titer achieved with a 40 µL inoculum (depicted in Figure 2A). In contrast, for the high-dose mice receiving 2000 PFU of virus, lung viral titers escalated in tandem with the incremented inoculation volumes. Notably, the apex of viral titers for both 20 µL and 40 µL inocula significantly deviated from that of the 10 µL counterpart (Figure 2B).

### 3.3. Lung Histopathological Changes in Mice Infected with Equivalent Influenza Virus Dosage at Different Volumes

**Figure 3:**
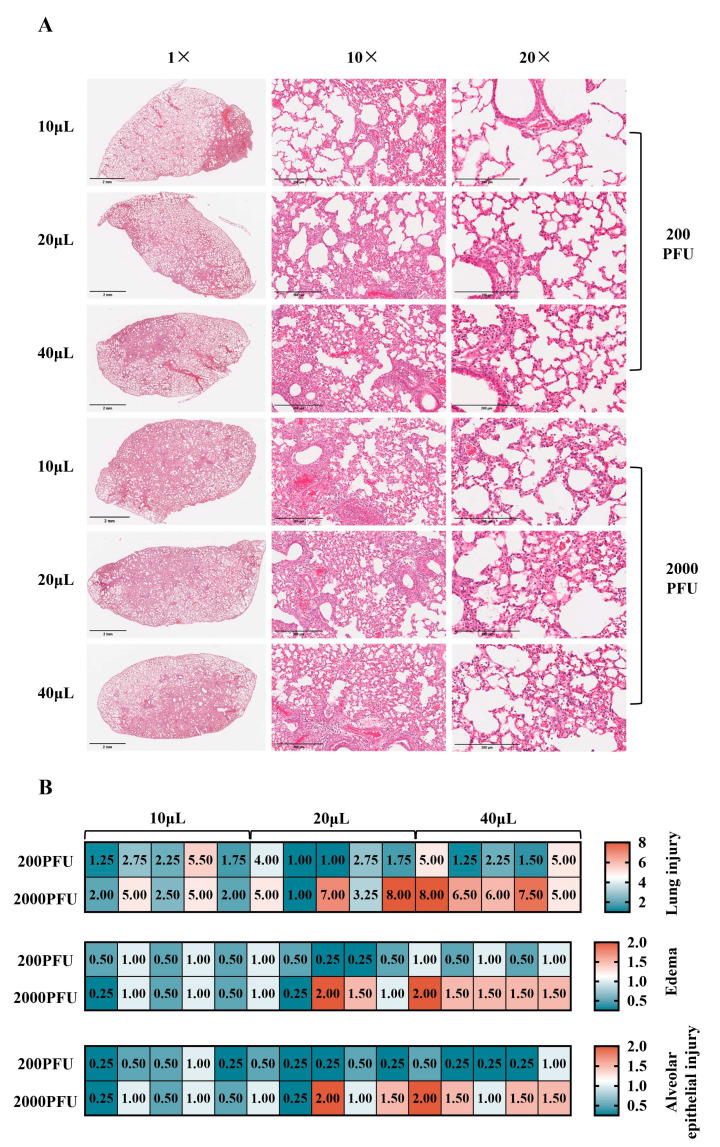
Lung histopathology of mice infected with different volumes of influenza virus at the same dose. Five mice were euthanized in batches at 1 day, 3 days, and 5 days post-infection. The left lung lobe tissues were collected, fixed with 4% paraformaldehyde, paraffin-embedded, sliced, and stained with hematoxylin and eosin, and images were scored in a double-blind manner. (**A**) Pulmonary histopathology. Overall view of tissue (1×, scale bar: 2 mm) shows borderline lesion between mild and severe lesion (10×, scale bar: 300 μm) and distal alveolar epithelial lesion (20×, scale bar: 200 μm). (**B**) Lung histological score. A semiquantitative analysis of lung injury, encompassing edema and alveolar epithelial damage, is articulated through a heat map representation.

To ascertain disparities in lung tissue pathological damage subsequent to infection with an equivalent dose of influenza A virus administered at varying inoculation volumes, we undertook histopathological analyses. The findings indicate that at Dpi5, within the 200 PFU virus infection cohort, both tissue damage and edema, along with distal alveolar epithelial cell injury, were less pronounced for the 10 μL and 20 μL inoculation volumes compared to the 40 μL volume, which exhibited more substantial pathological changes. In contrast, in the 2000 PFU virus infection group, escalating viral inoculum volumes led to augmented tissue edema and severe damage to distal alveolar epithelial cells, culminating in widespread lung pathology (Figure 3A). A comprehensive overview of histological scores is presented through a heat map representation (Figure 3B).

### 3.4. The Cy7.5 Dye Used to Simulate Different Volumes of Virus Inoculation Showed Differences in Respiratory Tract Distribution

To visualize the distribution of fluid within the respiratory tract following intranasal inoculation with varying volumes of viral suspension, we employed Cy7.5 dye as a surrogate marker for the viral solution. Mice were administered equal masses of Cy7.5 dye at different volumes, and subsequent imaging of both the whole body and lung tissues with trachea was conducted to observe the fluorescence distribution patterns.

**Figure 4:**
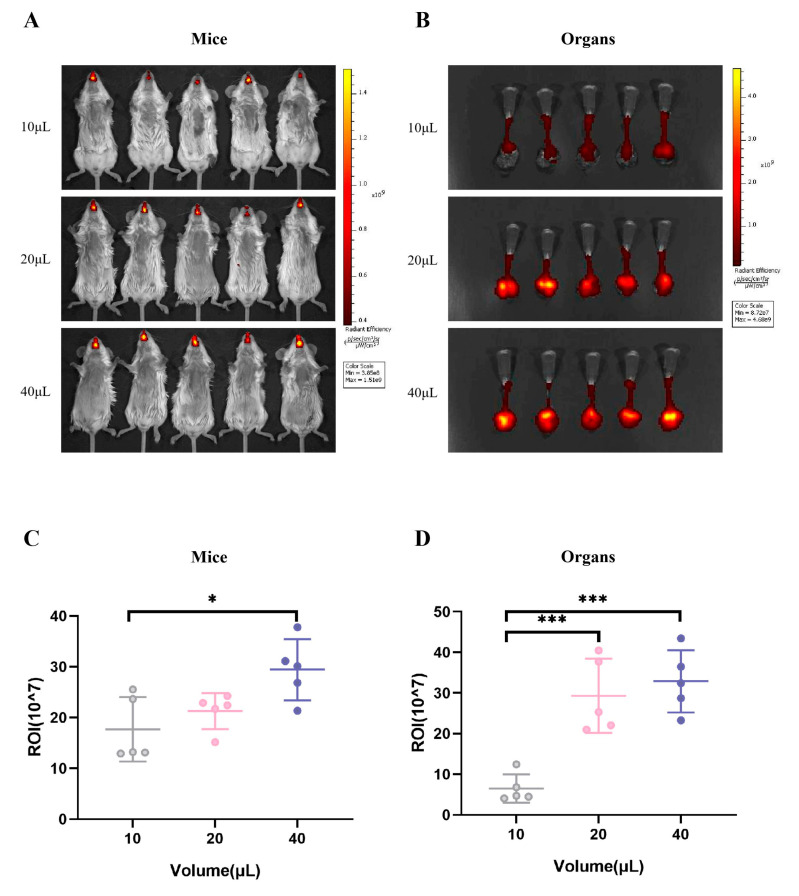
The fluorescence intensity of Cy7.5 dye within the murine respiratory tract was assessed following intranasal administration at volumes of 10 μL, 20 μL, and 40 μL, maintaining a constant mass for each inoculum. Mice were imaged in vivo 20 min after inoculation, and airway tissues, such as the trachea and lungs, were dissected for tissue-level imaging. Differences in the fluorescence intensity of Cy7.5 dye in the respiratory tract of mice at the same inoculation dose but different inoculation volumes were compared. (**A**,**C**) In vivo imaging and fluorescence intensity of mice. (**B**,**D**) In vitro tissue imaging and fluorescence intensity. * *p* < 0.05, , *** *p* < 0.005.

The imaging results from both whole-body and localized respiratory tissues demonstrated that the fluorescence intensity of Cy7.5 dye, when inoculated intranasally at different volumes but with the same mass, increased proportionally with the inoculation volume. In vivo imaging revealed a significant difference in fluorescence intensity between the 40 μL and 10 μL inoculation groups in the upper respiratory tract (Figure 4A,C). Furthermore, imaging of lung tissues with trachea indicated significantly higher fluorescence intensities for the groups inoculated with 20 μL and 40 μL Cy7.5 dye compared to the 10 μL group in the lower respiratory tract (Figure 4B,D).

## 4. Discussion

This study revealed that in low-dose infection groups, mice inoculated with a 40 μL viral volume exhibited a weight change trend significantly distinct from those inoculated with 10 μL and 20 μL viral volumes. Concurrently, the initial time to death in mice was observed to slow as the viral inoculation volume decreased. In contrast, within the high-dose infection group, both the weight change trajectory and mortality rates of mice administered 20 μL and 40 μL viral volumes were notably divergent from those given 10 μL. Furthermore, lung tissue virus titers across all groups mirrored the observed trends in weight variation and mortality, underscoring that variations in viral inoculation volume at an identical dose led to differential outcomes in viral replication among the groups. Of particular interest, the level of virus replication at a 20 μL inoculation volume demonstrated significant fluctuations across differing infection doses. Consequently, the precise selection of viral inoculum volume emerges as a critical determinant for the establishment of mouse models tailored to influenza virus investigations across diverse infection dosages.

At present, a limited number of studies have investigated the correlation between influenza virus infection and volume dependency. Notably, two studies have yielded contrasting conclusions. One study documented that in 10-week-old female mice, an inoculation volume smaller than 35 μL, when administered under a consistent infectious dose, resulted in a substantial decrease in mortality rates [31]. Conversely, another study found that intranasal infection in young mice (aged 2–4 months) with inoculation volumes of 20 μL and 40 μL did not significantly alter their mortality rates [35]. In this study, the results of the low-dose infection group were consistent with the former, while the results of the high-dose infection group were in line with the latter. This indicates that the volume dependency of influenza virus infection varies depending on the infectious dose. Therefore, when selecting appropriate mouse models for studying the pathogenesis of influenza and developing vaccines and drugs, it is necessary to comprehensively consider both the viral infection dose and the suitable volume of virus inoculation at that specific infection dose.

Under the same infectious dose, there are differences in the effect of virus infection among mice with different volumes of virus inoculation. Studies have shown that a potential mechanism for this difference is that when mice wake up from anesthesia, those inoculated with a lower volume may proportionally expel more virus from their respiratory tract [31]. This viewpoint was verified in the Cy7.5 dye mouse fluorescence experiment in this study. The fluorescence intensity of Cy7.5 dye in the lower respiratory tract was significantly higher with 20 μL and 40 μL inoculation volumes than with the 10 μL inoculation volume, indicating that a higher inoculation volume tends to enter the lower respiratory tract more easily compared to a lower inoculation volume. In the case of a constant total amount, the corresponding amount remaining in the upper respiratory tract should be higher with a lower volume than with a higher volume. However, the results of in vivo imaging in mice showed that the fluorescence intensity of Cy7.5 dye in the upper respiratory tract still increased with the increase of the inoculation volume, indicating that a 10 μL inoculation volume of Cy7.5 dye was expelled more from the respiratory tract compared to the 20 μL and 40 μL inoculation volumes. Based on this, it can be inferred that the volume of virus expelled from the respiratory tract by mice after waking up from anesthesia is similar. When the expelled volume of virus is constant, a lower volume of virus inoculation leaves proportionally less virus behind compared to a higher volume of virus inoculation. Therefore, at low infection doses, the effect of virus infection in mice with a 40 μL virus inoculation volume showed statistical differences compared to those with 10 μL and 20 μL virus inoculation volumes. At high infection doses, the effect of virus infection in mice with 20 μL and 40 μL virus inoculation volumes showed significant differences compared to that with a 10 μL virus inoculation volume.

Another possibility leading to this difference is the physiological variance in cellular microstructure between the upper and lower respiratory tracts. The upper respiratory tract comprises the nasal cavity, larynx, and trachea, whereas the lower respiratory tract includes the bronchi and lungs [36]. When the influenza virus enters the host, it directly invades cells in the upper respiratory tract. The cells in the upper respiratory tract mainly comprise epithelial cells and mucus gland cells in the nasal cavity and ciliated epithelial cells in the trachea. These cells exhibit polarization in two ways: ciliary polarization and mucous layer polarization. The polar arrangement of cilia forms an effective clearing mechanism via directional cooperative movements. The mucus layer traps and removes foreign pathogens and particles through components such as mucin [37,38,39]. In contrast, the lower respiratory tract is crucial for transporting air to the lungs and facilitating gas exchange. The upper cortical cells of the lower respiratory tract are diverse and lack a single structure with specific polarity [40]. Therefore, a higher viral inoculation volume may have more opportunities to introduce the influenza virus deeper into the lower respiratory tract compared to a lower viral inoculation volume. The physical barrier deep in the lower respiratory tract is less stringent than that in the upper respiratory tract, leading to disease aggravation. The fluorescence experiment using Cy7.5 in mice showed that the fluorescence intensity in the respiratory tract increased with an increase in the inoculation volume at the same dose. The fluorescence intensity in the lower respiratory tract for the 20 μL and 40 μL inoculation volumes was significantly higher than that for the 10 μL volume. Previous studies have shown that using radioactive tracers in mouse models to monitor the distribution of substances administered as nasal drops revealed that most tracers are located in the upper respiratory tract at low volumes. As the volume of nasal drops increases to ≥35 μL, an increasing number of radioactive tracers are detected in the lower respiratory tract [30,41].

## 5. Conclusions

In this study, we analyzed the effects of different viral infection volumes on the effectiveness of virus infection in mice at the same dose and the variations of these effects across different doses. We found that even under the same infection dose, different inoculation volumes can lead to differences in pathogenicity outcomes. Of particular note, in various infection dose regimens, the viral replication efficacy at a 20-microliter inoculation volume demonstrated significant fluctuations, indicating that the volume dependence of viral infection varies with different infection doses. These results will help other researchers choose the appropriate viral infection volume when establishing mouse models for studying the pathogenesis of influenza and developing vaccines and drugs.

## Data Availability

The data presented in this paper are available on request from the corresponding author.
